# Differential targeting of the cyclin-dependent kinase inhibitor, p21^CIP1/WAF1^, by chelators with anti-proliferative activity in a range of tumor cell-types

**DOI:** 10.18632/oncotarget.5088

**Published:** 2015-08-22

**Authors:** Rayan S. Moussa, Zaklina Kovacevic, Des R. Richardson

**Affiliations:** ^1^ Molecular Pharmacology and Pathology Program, Discipline of Pathology and Bosch Institute, Blackburn Building (D06), The University of Sydney, Sydney, New South Wales, 2006, Australia

**Keywords:** DFO, Dp44mT, p21^CIP1/WAF1^, p53, MDM2

## Abstract

Chelators such as 2-hydroxy-1-napthylaldehyde isonicotinoyl hydrazone (311) and di-2-pyridylketone-4,4-dimethyl-3-thiosemicarbazone (Dp44mT) target tumor cell iron pools and inhibit proliferation. These agents also modulate multiple targets, one of which is the cyclin-dependent kinase inhibitor, p21. Hence, this investigation examined the mechanism of action of these compounds in targeting p21. All the chelators up-regulated *p21* mRNA in the five tumor cell-types assessed. In contrast, examining their effect on total p21 protein levels, these agents induced either: (1) down-regulation in MCF-7 cells; (2) up-regulation in SK-MEL-28 and CFPAC-1 cells; or (3) had no effect in LNCaP and SK-N-MC cells. The nuclear localization of p21 was also differentially affected by the ligands depending upon the cell-type, with it being decreased in MCF-7 cells, but increased in SK-MEL-28 and CFPAC-1 cells. Further studies assessing the mechanisms responsible for these effects demonstrated that p21 expression was not correlated with p53 status, suggesting a p53-independent mechanism. Considering this, we examined proteins that modulate p21 independently of p53, namely NDRG1, MDM2 and ΔNp63. These studies demonstrated that a dominant negative MDM2 isoform (p75^MDM2^) closely resembled p21 expression in response to chelation in three cell lines. These data suggest MDM2 may be involved in the regulation of p21 by chelators.

## INTRODUCTION

Iron (Fe) deprivation *via* the use of chelators, has been shown to lead to G_1_/S arrest in neoplastic cells [1–7]. In fact, cellular Fe levels modulate the expression of molecules involved in cell cycle control, including cyclins, cyclin-dependent kinases (cdk), cdk inhibitors, as well as tumor suppressor and metastasis suppressor genes [8–12]. Since neoplastic cells have a greater need for Fe, they are more susceptible to the effects of Fe chelation when compared to normal cells [13, 14]. Thus, by inhibiting Fe availability to tumors, cancer cell proliferation can be effectively blocked, indicating that targeting Fe and other essential metals is a significant new therapeutic strategy [9, 15, 16].

**Figure 1:**
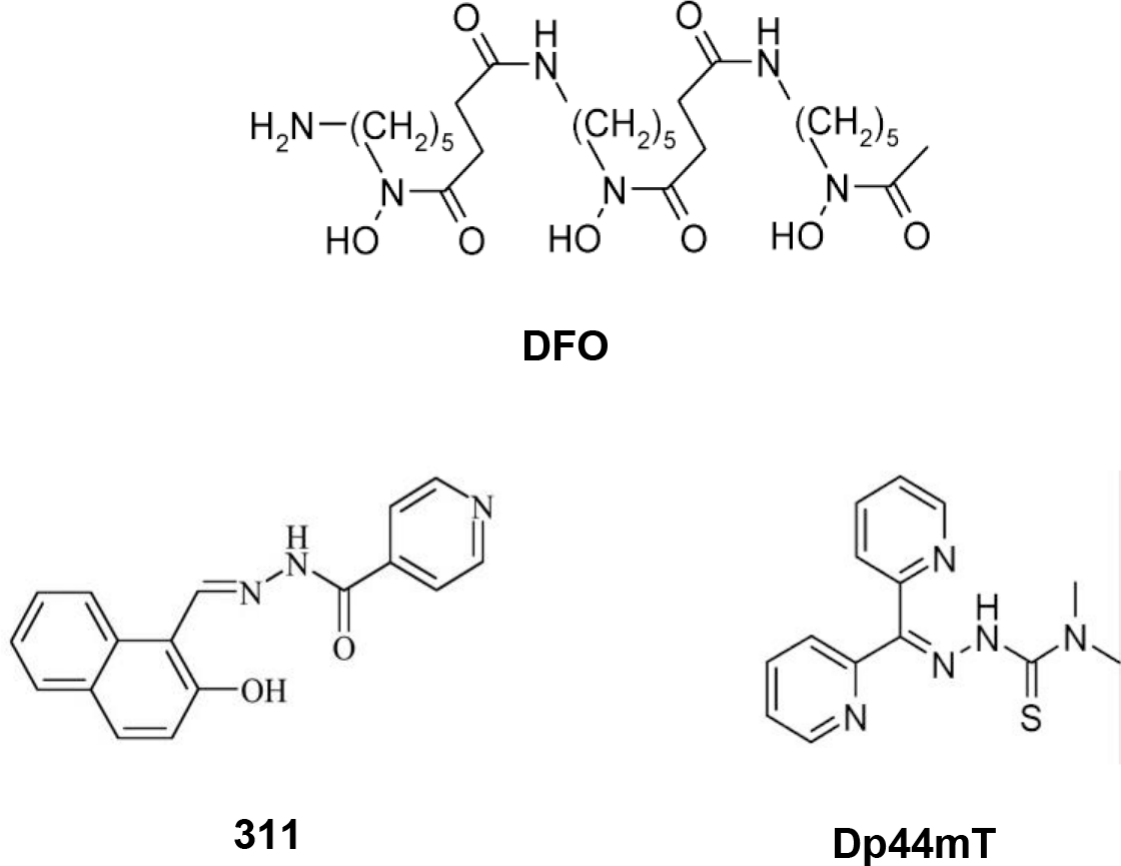
Line drawings of the structures of the chelators: DFO, 311 and Dp44mT

Desferrioxamine (DFO; Fig. 1) is a well known Fe chelator that is clinically used for the treatment of the Fe overload disease, β-thalassemia [15]. The potential of chelators as anti-cancer agents was realised when DFO was trialled in a number of *in vitro* and *in vivo* studies, some of which showed promising results [1, 4, 5, 17]. Although DFO has shown anti-proliferative activity, the high hydrophilicity of this ligand limits its membrane permeability and anti-tumor efficacy [18, 19]. As a result of this problem and its short half-life in the circulation, DFO must be administered *via* subcutaneous infusion for extensive periods, making it inconvenient for patients [15].

Due to the limitations of DFO, alternative chelators have been developed in the quest to create more potent and selective anti-cancer agents [15]. For example, 2-hydroxy-1-napthylaldehyde isonicotinoyl hydrazone (311; Fig. 1), is a ligand of the pyridoxal isonicotinoyl hydrazone (PIH) class that has been shown to be more effective at chelating cellular Fe than DFO, and this can be explained by its greater lipophilicity [20–22]. Furthermore, di-2-pyridylketone 4,4-dimethyl-3-thiosemicarbazone (Dp44mT; Fig. 1), is a novel agent from the dipyridyl thiosemicarbazones (DpT) class of chelators that has been demonstrated to have markedly greater anti-proliferative activity and Fe chelation efficacy than DFO and is highly effective at reducing growth and metastasis of multiple tumors *in vitro* and *in vivo* [23–26]. An important aspect of the activity of the DpT class of thiosemicarbazones (*e.g*., Dp44mT) is their ability to: **(1)** up-regulate the potent metastasis suppressor, N-myc downstream regulated gene-1 (NDRG1) [12, 25], which inhibits the epithelial mesenchymal transition [27] through its effect on a number of oncogenic signaling pathways [14, 27–31]; and **(2)** their ability to overcome multi-drug resistance [24] by causing lysosomal membrane permeabilization *via* their metal-induced redox activity [32, 33]. In fact, the DpT series of ligands form redox-active complexes with Fe and copper, which leads to the generation of reactive oxygen species (ROS) that enhance cellular toxicity [33–35].

In order to develop these agents further, and to better understand their mechanisms of action, the effects of chelators on the expression of cell cycle control molecules require further elucidation. In fact, apart from up-regulating NDRG1, chelators were found to affect a number of crucial molecules that are involved in proliferation and apoptosis [36]. Among these, cellular Fe chelation up-regulates the expression and transcriptional activity of the tumor suppressor, p53 [10, 37]. Additionally, Fe depletion increased p53 phosphorylation, which stabilizes the p53 protein, preventing its proteasomal degradation [38]. Notably, p53 plays a key role in regulating the expression of genes involved in cell cycle arrest and apoptosis in response to genotoxic damage or cellular stress [39].

Thus, NDRG1 and p53 are important molecular targets of chelators, which are crucial to the anti-cancer and anti-metastatic activity of these agents [10, 12, 16]. These molecules and their downstream protein targets present ideal therapeutic strategies for the treatment of cancer. In fact, the cdk inhibitor, p21, is a common downstream target for both p53 [40] and NDRG1 [41] and plays an important role in the inhibition of cell cycle progression and proliferation [42, 43], as well as prevention of metastasis [44].

Significantly, p21 plays a variety of physiological roles, many of which rely on its nuclear localization [45]. These include its cdk inhibitory function, promotion of differentiation and of cellular senescence [45]. For instance, p21 inhibits the cyclin D/cdk4/6 and the cyclin E/cdk2 complexes in response to DNA-damage, resulting in G_1_/S arrest [46]. In addition, p21 also binds to the DNA replication/repair factor, proliferating cell nuclear antigen (PCNA), interfering with its DNA replication, but not DNA repair activity [47, 48]. Paradoxically, when localized (at least initially) to the cytosol, p21 can aid cell cycle progression and play vital pro-proliferative and cell survival roles [45]. At low levels, p21 can aid cell cycle progression, by stabilizing interactions between cdk4/6 and cyclin D1 [49, 50]. Notably, p21 also has anti-apoptotic functions when up-regulated in tumor cells [51]. Considering its crucial role [46, 49], it is vital to elucidate how p21 responds to Fe-depletion in various cancer cells and whether it can be utilized as a therapeutic target.

The current study investigated the mechanisms involved in the chelator-mediated regulation of p21. Five cancer cell lines with different p53 status were incubated with either of three ligands, namely: DFO, 311 or Dp44mT. In addition, the well characterized DNA-damaging agents: actinomycin D (Act D), cisplatin (CP) and mitomycin C (MC) [52–55], were also used throughout this study as positive controls. Hence, for the first time, the effect of a variety of effective chelators could be assessed relative to a range of DNA-damaging agents to assess the mechanism involved. These results revealed a number of significant outcomes, namely: (**1**) the effect of the chelators on p21 differed between the cell-types examined, with p21 protein being up-regulated in SK-MEL-28 and CFPAC-1 cells, down-regulated in MCF-7 cells, and left unchanged in LNCaP and SK-N-MC cells; (**2**) the nuclear localization of p21 was also differentially affected by the ligands depending upon the cell-type, with it being decreased in MCF-7 cells, but increased in SK-MEL-28 and CFPAC-1 cells; (**3**) the p53 status of the cells examined showed no correlation to the chelator-mediated effects on p21, suggesting a p53-independent effect; and (**4**) examination of molecules that can modulate p21 independently of p53, namely NDRG1 [41], mouse double minute 2 homolog (MDM2) [56, 57] and ΔNp63 [58–61], revealed that a dominant negative MDM2 isoform (p75^MDM2^) closely resembled p21 expression in response to chelation in three of the five cell lines examined. In fact, the p75^MDM2^ isoform has been shown to interfere with the ability of the full-length MDM2 (p90^MDM2^) to degrade its target proteins [62], which include p53 and p21 [56, 57, 63, 64]. These data suggest that MDM2 may be one effector through which chelators regulate p21 expression in these cell-types.

## RESULTS

### Incubation with chelators or DNA-damaging agents up-regulates p21 mRNA expression

**Figure 2:**
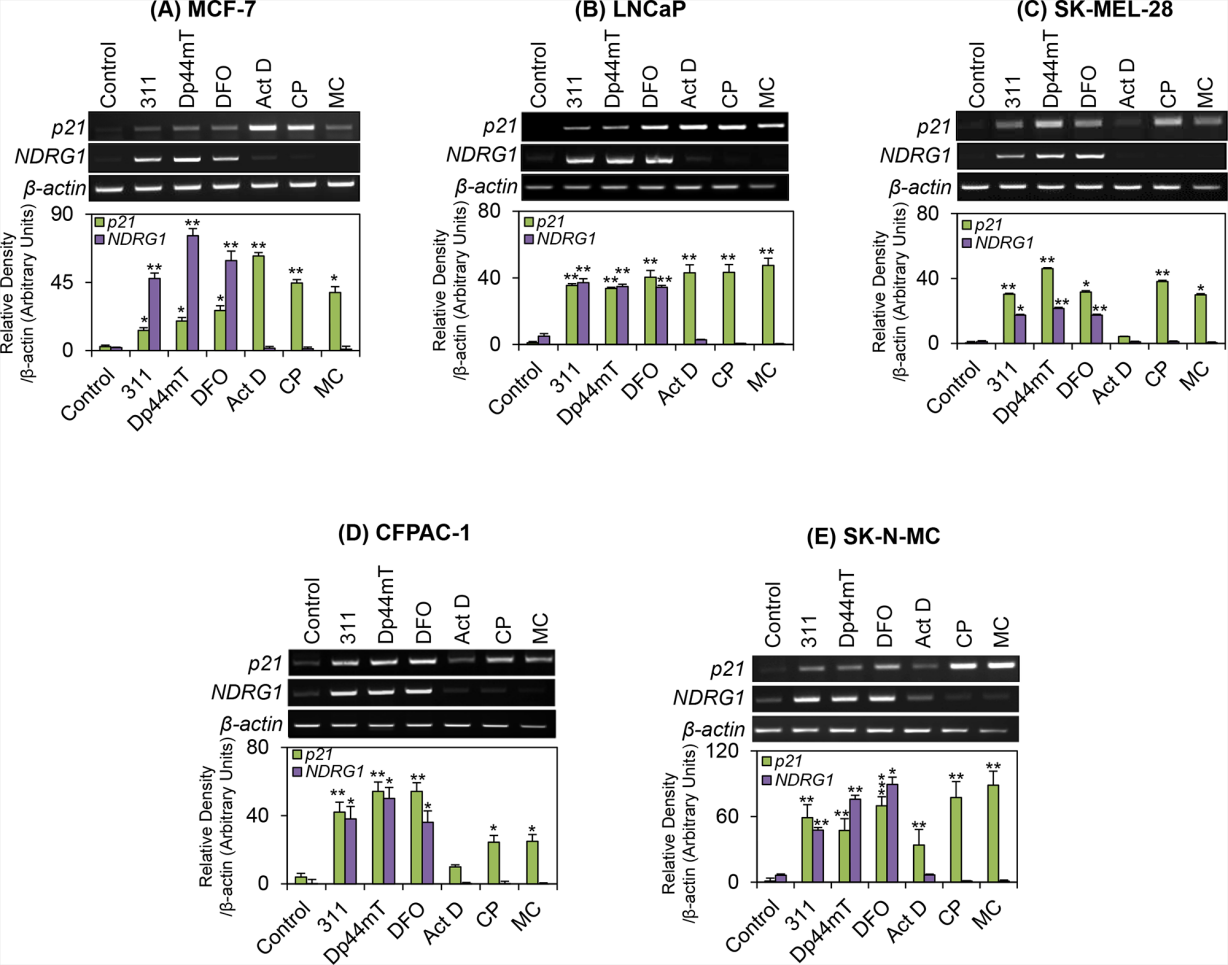
The effect of the chelators 311, Dp44mT, or DFO, and the DNA-damaging agents, Act D, CP or MC on *p21* or *NDRG1* mRNA levels in five different tumor cell lines **A.** MCF-7; **B.** LNCaP; **C.** SK-MEL-28; **D.** CFPAC-1; and **E.** SK-N-MC cells. Cells were incubated for 24 h/37°C with the chelators, 311 (25 μM), Dp44mT (2.5 μM), DFO (250 μM), or the DNA-damaging agents, Act D (5 nM), CP (20 μM), or MC (30 μM). The gels are typical of 3 independent experiments, while the densitometric analysis is mean ± SD (3 experiments). Relative to control: *p < 0.05, **p < 0.01, ***p < 0.001.

To examine the effect of chelators on p21 expression, our initial studies focused on examining *p21* mRNA levels in five different cell-types, namely: MCF-7, LNCaP, SK-MEL-28, CFPAC-1 and SK-N-MC cells (Fig. 2A–2E). These cell lines were specifically chosen due to their varying p53 status (Table 1), with MCF-7 and LNCaP cells expressing wild-type (WT) p53, while SK-MEL-28 and CFPAC-1 cells express mutant p53, and SK-N-MC cells are p53 null [65–69]. Considering that p53 is an important transcriptional activator of p21 [39], we initially hypothesised that p21 expression could correlate to p53 status and activity in the cells examined.

**Table 1:** A summary of p21 mRNA and protein expression in response to Fe chelators or DNA-damaging agents

p53 status	Cell line	Untreated Control		General effect of Fe Chelators		General effect of DNA-damaging agents	
		*p21* mRNA	p21 protein	*p21* mRNA	p21 protein	*p21* mRNA	p21 protein
**WT p53^1–3^**	MCF-7	Little expression	+	+	-	+	+**
	LNCaP	Little expression	+	+	No change vs. Control	+	+
**Mutant p53^1,4^**	SK-MEL-28	Little expression	No expression	+	+	+*	No expression
	CFPAC-1	Little expression	+	+	+	+*	No change vs. Control
**p53 Null^5^**	SK-N-MC	No expression	+	+	No change vs. Control	+	No change vs. Control

1 O'Connor *et. al.* Cancer Res. 1997; 57: 4285–4300.

2 Wosikowski *et. al.* Cell Growth Differ. 1995; 6: 1395–1403.

3 Jackson *et. al.* Urol Oncol. 2002; 7: 99–104.

4 Redston *et. al.* Cancer Res. 1994; 54: 3025–3033.

5 Moll *et. al*. Mol Cell Biol. 1996; 16: 1126–1137.

* Unlike CP and MC, Act D did not increase *p21* mRNA in these cell lines relative to the control (Fig. 2C and 2D).

**Figure 3:**
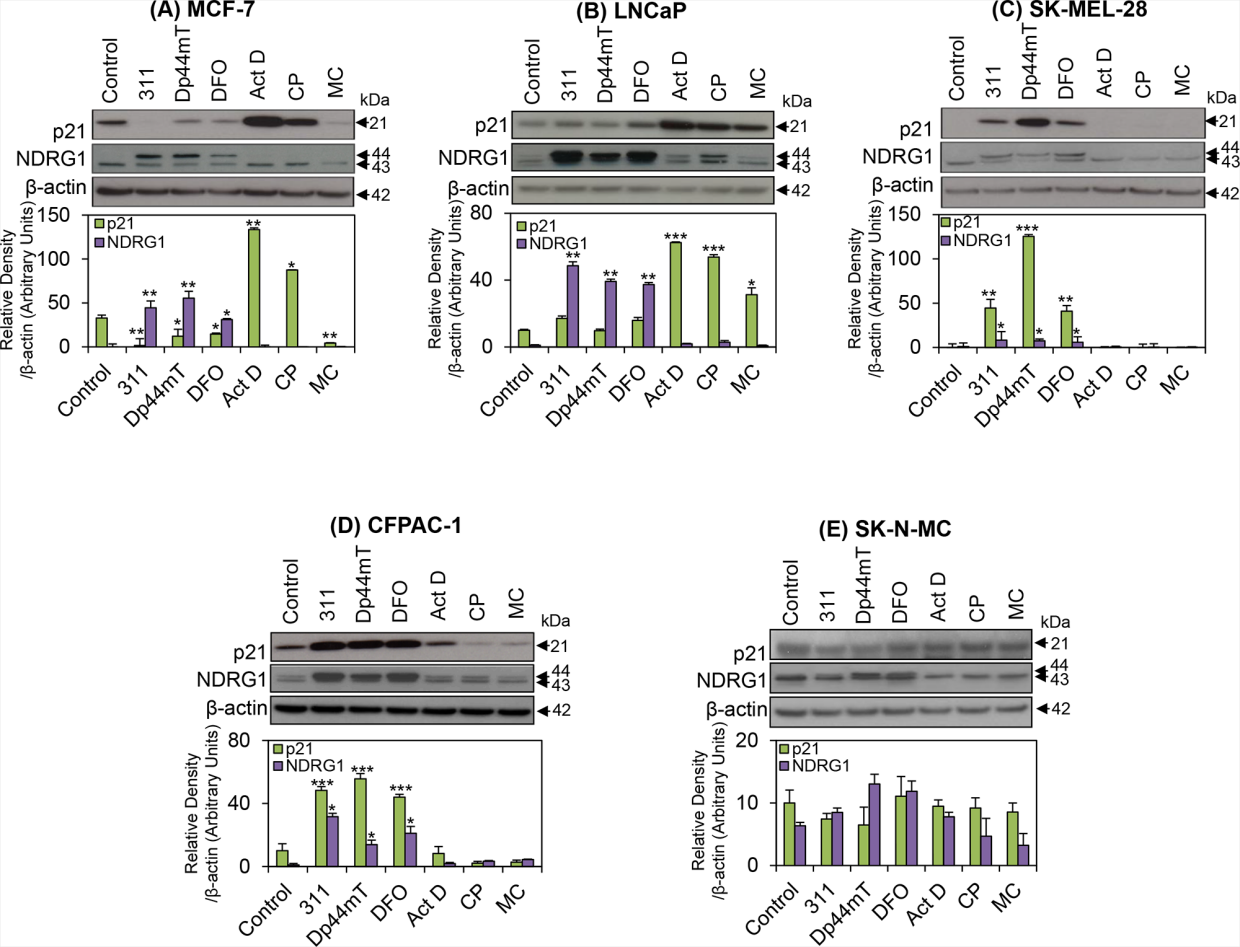
The effect of the chelators 311, Dp44mT, or DFO, and the DNA-damaging agents, Act D, CP or MC on p21 or NDRG1 protein levels in five different tumor cell lines **A.** MCF-7; **B.** LNCaP; **C.** SK-MEL-28; **D.** CFPAC-1; and **E.** SK-N-MC cells. Cells were incubated for 24 h/37°C with the chelators, 311 (25 μM), Dp44mT (2.5 μM), DFO (250 μM), or the DNA-damaging agents, Act D (5 nM), CP (20 μM), or MC (30 μM). The blots are typical of 3–6 independent experiments, while the densitometric analysis is mean ± SD (3–6 experiments). Relative to control: *p < 0.05, **p < 0.01, ***p < 0.001.

** Unlike Act D and CP, MC significantly decreased p21 protein levels in this cell line relative to the control (Fig. 3A).

In these studies, a 24 h incubation with the chelators, 311 (25 μM), Dp44mT (2.5 μM) and DFO (250 μM) was utilized. These concentrations were used due to the relative permeability and efficacy of these ligands. Indeed, DFO is highly hydrophilic and does not permeate cell membranes effectively [19], while 311 and Dp44mT are both lipophilic and easily permeate cell membranes to chelate intracellular Fe pools [21, 23]. A lower concentration of Dp44mT was used relative to 311 due to the markedly greater anti-proliferative activity of the former compound [21, 23]. These concentrations also allowed comparisons to the results obtained in previous studies using these agents [14, 70]. After all incubations at these concentrations, the cell monolayer remained intact and the cells were viable.

Using these experimental conditions with the chelators, a significant (*p* < 0.001–0.05) up-regulation in *p21* mRNA levels was observed across all cell-types examined when compared to untreated control cells (Fig. 2A–2E). The DNA-damaging agents, Act D (5 nM), CP (20 μM) and MC (30 μM), were used as positive controls, as these conditions are known to markedly up-regulate *p21* mRNA in MCF-7 cells [12]. Following a 24 h incubation, both CP and MC significantly (*p* < 0.01–0.05) up-regulated *p21* mRNA levels in each of the cell-types examined (Fig. 2A–2E). In contrast, while Act D increased *p21* mRNA relative to the control, the effect was variable between cell-types, with no marked up-regulation occurring for SK-MEL-28 (Fig. 2C) and CFPAC-1 (Fig. 2D). On the other hand, a marked and significant (*p* < 0.01) up-regulation of *p21* mRNA occurred when MCF-7, LNCaP and SK-N-MC cells were treated with Act D relative to the control (Fig. 2A, 2B, 2E).

A well characterised molecular target of Fe depletion, namely *NDRG1* [12, 25], was also used as a positive control for Fe-mediated regulation by the chelators. As shown in Fig. 2A–2E, in all five cell-types the chelators resulted in a significant (*p* < 0.01–0.05) increase in *NDRG1* mRNA levels relative to the control, which is in agreement with earlier studies [12]. The DNA-damaging agents displayed no significant (*p* > 0.05) effects on *NDRG1* expression in these cell lines.

In summary, in each of the cell lines examined, the chelators were able to markedly up-regulate *p21* mRNA levels, regardless of the p53 status of the cell (Table 1). These results suggest that the chelators may regulate *p21* mRNA independently of p53 status.

### p53-independent regulation of p21 protein in response to Fe chelators

Previous studies [70, 71] demonstrated that although DFO or 311 increased *p21* mRNA expression in MCF-7 cells, the protein levels of p21 were markedly reduced by these agents. To further investigate this, the next series of studies focused on examining the protein levels of p21 in all five cell lines. Under control conditions, MCF-7 cells expressed relatively high levels of p21 protein (Fig. 3A). In agreement with previous findings [70, 71], the protein levels of p21 were significantly (*p* < 0.01–0.05) decreased by the chelators in this cell line, when compared to the control (Fig. 3A). This effect was in contrast to the up-regulation of *p21* mRNA levels that was observed after incubation of MCF-7 cells with these agents relative to control medium (Fig. 2A). However, the DNA-damaging agents, Act D and CP, significantly (*p* < 0.01–0.05) increased the levels of p21 protein in MCF-7 cells (Fig. 3A), which was in agreement with their effect on *p21* mRNA expression (Fig. 2A). The effect of Act D and CP on increasing p21 protein was expected based on the fact that MCF-7 cells express wild-type p53 which responds to DNA damage [39] (Table 1). However, unexpectedly, treatment of MCF-7 cells with the DNA-damaging agent, MC, resulted in a significant (*p* < 0.01) decrease in p21 protein expression relative to the control (Fig. 3A).

In contrast to MCF-7 cells, incubation of LNCaP cells with 311, Dp44mT, or DFO, failed to significantly (*p* > 0.05) alter p21 protein levels relative to the control (Fig. 3B). However, incubating LNCaP cells with the DNA-damaging agents, Act D, CP, or MC, also resulted in a marked and significant (*p* < 0.001–0.05) increase in p21 levels relative to the control (Fig. 3B). Considering that LNCaP cells also express wild-type p53 (Table 1; [65]), the observed effect of DNA-damaging agents on the up-regulation of p21 expression was expected.

In contrast to MCF-7 and LNCaP cells, using SK-MEL-28 and CFPAC-1 cells, chelators markedly (*p* < 0.001–0.01) increased p21 levels in comparison to the untreated controls (Fig. 3C, 3D). However, relative to the control, there were no significant (*p* > 0.05) alterations in p21 protein levels following incubation with the DNA-damaging agents in both these cell lines, and this could be due to the fact that they possess mutated p53 [66, 68]. Clearly, this response to chelation and DNA-damaging agents by SK-MEL-28 (Fig. 3C) and CFPAC-1 cells (Fig. 3D) is different to that observed for MCF-7 and LNCaP cells (Fig. 3A).

Examining SK-N-MC cells, p21 was markedly expressed in untreated control cells (Fig. 3E). Following incubation with 311 or Dp44mT, there was a slight, but not significant (*p* > 0.05) decrease in p21 protein levels in comparison to control cells, while DFO exerted no significant effect (Fig. 3E). In addition, incubation with DNA-damaging agents resulted in no significant (*p* > 0.05) change in p21 protein expression relative to the control in SK-N-MC cells (Fig. 3E), which was expected considering that these cells are p53 null [69]. In contrast to the results with the other cell-types, the results in terms of p21 protein expression obtained with SK-N-MC cells varied between investigations. For instance, during the current study, incubation with chelators resulted in a consistently slight, but not significant (*p* > 0.05) decrease in p21 expression in SK-N-MC cells over 6 experiments (Fig. 3E). On the other hand, several previous investigations reported a slight increase of p21 protein relative to the control [9], or significant up-regulation [72]. This variability in response of p21 to the agents was only observed for SK-N-MC cells and may be related to clonal differences in tumor cell lines that occur as a function of passage number [73].

Considering that the metastasis suppressor NDRG1 was found to up-regulate p21 expression in a wide variety of cancer cells [41], we further examined the levels of NDRG1 in response to the Fe chelators and DNA damaging agents in each of the five cell lines. As previously reported [25], two NDRG1 bands at 43- and 44-kDa were detected in all cell lines examined (Fig. 3A–3E). These bands may correspond to different post-translational modifications *e.g*., truncation and phosphorylation states of NDRG1 [74, 75]. Hence, the densitometric analysis performed for NDRG1 herein represents the sum of the two bands. As shown in Fig. 3A–3D, NDRG1 protein levels were significantly (*p* < 0.01–0.05) increased in MCF-7, LNCaP, SK-MEL-28 and CFPAC-1 cells after incubation with the chelators. A slight, but not significant (*p* > 0.05) increase in NDRG1 was also observed in SK-N-MC cells following incubation with these ligands. However, only in SK-MEL-28 (Fig. 3C) and CFPAC-1 cells (Fig. 3D) did the expression of NDRG1 directly correlate with increased p21 levels after incubation with these latter agents. No significant (*p* > 0.05) alterations in the expression of NDRG1 protein levels were detected after incubation with the DNA-damaging agents relative to the control in all cell-types (Fig. 3A–3E).

Since p53 plays an important role in sensing both nutrient-depletion and DNA-damage [11, 76, 77], and can directly influence p21 expression [40], the difference in the response of p21 protein expression to chelators and DNA-damaging agents was hypothesized to be due to differences in p53 status between the cell-types (Table 1). However, collectively, assessing all cell-types examined, increased *p21* mRNA and protein expression after incubation with chelators was not positively correlated to WT p53 status (Table 1). This conclusion is clear when comparing the effect of chelators on MCF-7 cells and SK-MEL-28 cells (Fig. 3A, 3C). In fact, MCF-7 cells have WT p53 and demonstrated a decrease in p21 protein after incubation with chelators (Fig. 3A; Table 1), while SK-MEL-28 cells are p53 mutant and showed a marked increase in p21 protein after treatment with these agents (Fig. 3C; Table 1).

It should be noted that the three chelators used in this investigation increased *p21* mRNA levels in all cell-types examined (Fig. 2A–2E). In contrast, the protein levels were significantly (*p* < 0.01–0.05) decreased by these agents in MCF-7 cells (Fig. 3A). These observations suggest that p21 translation may be inhibited, or alternatively, there may be increased p21 protein degradation in this cell-type. Indeed, inhibition of protein synthesis is known to occur after incubation of cells with chelators, including DFO and 311 [21].

### The expression of the MDM2 oncoprotein in response to chelators may affect expression of p21

**Figure 4:**
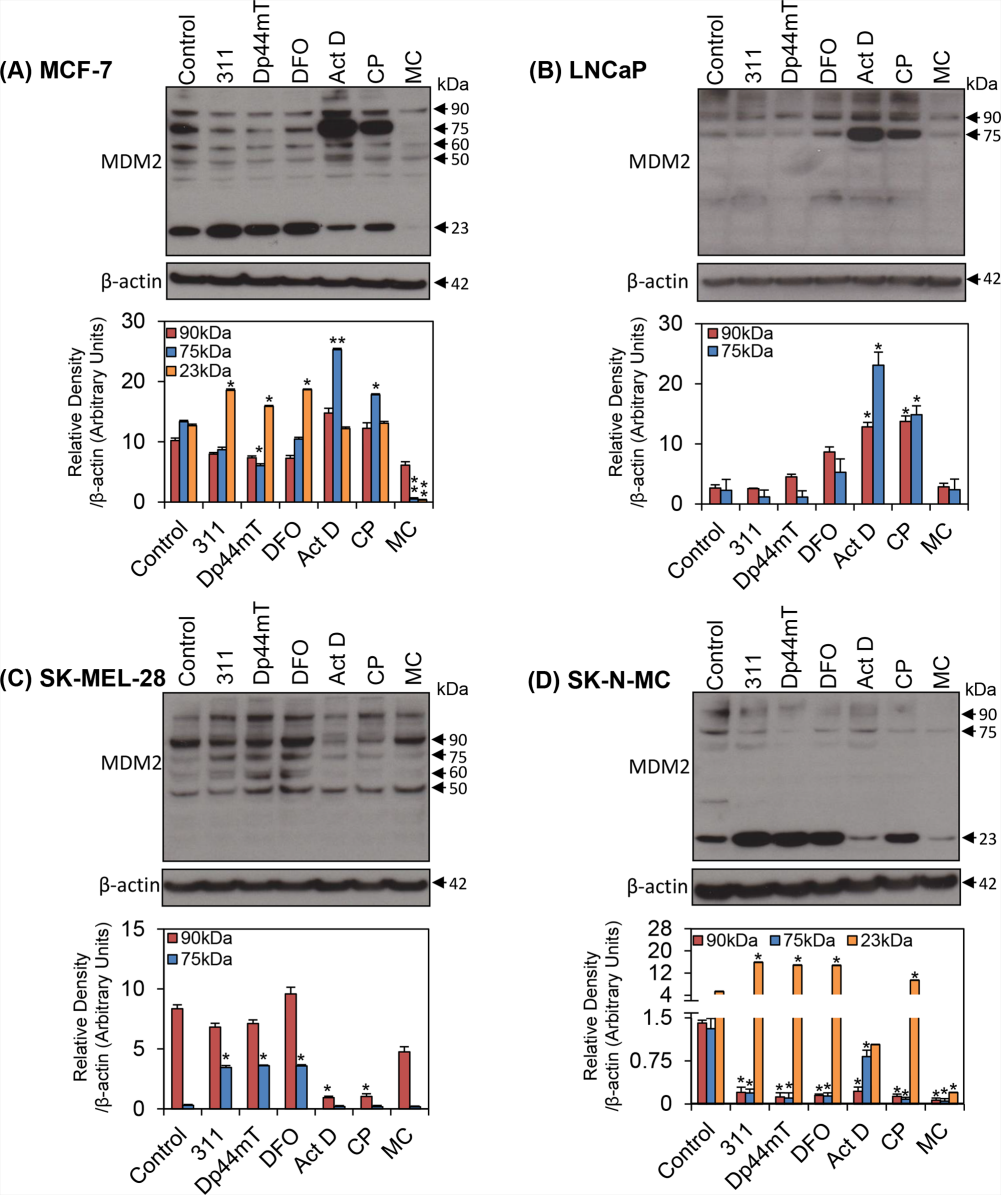
The effect of the chelators 311, Dp44mT, or DFO, and the DNA-damaging agents, Act D, CP or MC on MDM2 protein isoforms in four different tumor cell lines **A.** MCF-7; **B.** LNCaP; **C.** SK-MEL-28; and **D.** SK-N-MC cells. Cells were incubated for 24 h/37°C with the chelators, 311 (25 μM), Dp44mT (2.5 μM), DFO (250 μM), or the DNA-damaging agents, Act D (5 nM), CP (20 μM), or MC (30 μM). The blots are typical of 3 independent experiments, while the densitometric analysis is mean ± SD (3 experiments). Relative to control: *p < 0.05, **p < 0.01, ***p < 0.001.

As p53 status between the cell-types examined did not satisfactorily explain the different p21 response to the chelators, further studies were performed to determine the molecular mechanisms involved. Considering this, studies then assessed MDM2, which functions as an oncoprotein and a negative regulator of both p53 and p21 [56, 57, 63, 64]. Interestingly, MDM2 has also been shown to promote p21 protein degradation independently of its effects on p53 [56, 57]. Hence, the following investigation focused on the effect of the agents on MDM2 expression (Fig. 4A–3D). Numerous isoforms of MDM2 are known to exist (*i.e*., full-length p90^MDM2^, p75^MDM2^, p60^MDM2^, p50^MDM2^, *etc*.), each having various effects on its downstream targets [78, 79]. Two particular isoforms, namely p90^MDM2^ and p75^MDM2^, have been reported to predominate in both human and murine cells [80, 81]. The over-expression of p75^MDM2^, which cannot bind to p53, has been found to interfere with the ability of p90^MDM2^ to promote p53 degradation [62]. Hence, the effects of the agents on these two latter isoforms were particularly important to assess.

First, it is important to note that despite exhaustive attempts, western blot analysis revealed that of the five cell-types used, MDM2 isoforms were not detected in CFPAC-1 cells. This was in direct contrast to the other cell lines examined, and hence, only MDM2 blots for MCF-7 (Fig. 4A), LNCaP (Fig. 4B), SK-MEL-28 (Fig. 4C) and SK-N-MC cells (Fig. 4D) are shown. After incubation of MCF-7 cells with chelators, the expression of multiple isoforms of MDM2 was slightly, but not significantly (*p* > 0.05) decreased in comparison to the control cells. These included the p50^MDM2^, p60^MDM2^ and p90^MDM2^ isoforms (Fig. 4A). On the other hand, incubation of these cells with Dp44mT demonstrated a significant (*p* < 0.05) decrease in the expression of the p75^MDM2^ isoform. In contrast, incubation with Act D or CP resulted in a significant (*p* < 0.01–0.05) increase in the expression of the p75^MDM2^ isoform (Fig. 4A). However, MC significantly (*p* < 0.01) decreased the expression of the p75^MDM2^ band. Of relevance, the expression pattern of p75^MDM2^ was similar to that observed for p21 protein in this cell-type (Fig. 3A). Considering this relative to the ability of p75^MDM2^ to inhibit the function of p90^MDM2^ [62] to promote p21 degradation [56, 57], lower levels of p75^MDM2^ relative to the control would, in theory, promote lower p21 protein levels in MCF-7 cells, as shown in Fig. 3A.

A prominent band at approximately 23 kDa was also detected with the MDM2 antibody in MCF-7 cells (Fig. 4A), but does not correlate to any previously reported MDM2 isoform [78], and its functional significance remains unclear. Incubation of cells with chelators significantly (*p* < 0.05) increased expression of the 23 kDa band, whereas treatment with the DNA-damaging agent MC significantly (*p* < 0.01) reduced its expression (Fig. 4A). Considering these latter observations, this low *M_r_* band could potentially be a cleaved product of full-length p90^MDM2^, or its shorter isoforms. Notably, this 23 kDa band was consistently detected only in MCF-7 cells (Fig. 4A) and SK-N-MC cells (Fig. 4D), but not the other cell lines, indicating its expression was cell-type specific.

Assessing LNCaP cells, only the p75^MDM2^ and p90^MDM2^ isoforms were apparent (Fig. 4B). Densitometric analysis demonstrated that over 3 experiments, 311 and Dp44mT had no significant (*p* > 0.05) effect on the expression of both p75^MDM2^ and p90^MDM2^, while DFO caused a slight, but not significant (*p* > 0.05) increase in the expression of these isoforms relative to the control (Fig. 4B). The response of these isoforms to DNA-damaging agents in LNCaP cells (Fig. 4B) was similar to that found for MCF-7 cells (Fig. 4A), there being a significant (*p* < 0.05) increase in the p75^MDM2^ and p90^MDM2^ isoforms in response to the DNA-damaging agents, Act D and CP. On the other hand, MC had little effect relative to the control in LNCaP cells (Fig. 4B). It is evident that the expression patterns of the proteins, p21 (Fig. 3B) and p75^MDM2^ (Fig. 4B), was similar in LNCaP cells incubated with chelators, or the DNA-damaging agents, Act D or CP. Notably, a band above 90 kDa was also found in this cell-type (Fig. 4B), but does not correlate to any previously reported MDM2 isoforms [78], and was not consistently observed. Therefore, it may be attributed to non-specific binding of the MDM2 antibody. Furthermore, a band which appeared at approximately 37 kDa (Fig. 4B) was not always observed in repeat studies, and thus, was not considered further.

Four MDM2 isoforms were identified in SK-MEL-28 cells, namely p50^MDM2^, p60^MDM2^, p75^MDM2^ and p90^MDM2^ (Fig. 4C). Incubation of SK-MEL-28 cells with all chelators led to a significant (*p* < 0.05) increase in p75^MDM2^ relative to the control. This expression pattern was similar to that observed for p21 in this cell-type (Fig. 3C). On the other hand, the expression of p50^MDM2^, p60^MDM2^ or p90^MDM2^ was not affected by the chelators in these cells when compared to untreated controls (Fig. 4C). Incubation of these cells with DNA-damaging agents led to no significant (*p* > 0.05) alterations in the levels of p50^MDM2^, p60^MDM2^ or p75^MDM2^. In contrast, following Act D and CP treatment, there was a significant (*p* < 0.05) reduction in the expression of the p90^MDM2^ isoform. Once again, a non-specific band above 90 kDa was detected.

Examining SK-N-MC cells, both the p75^MDM2^ and p90^MDM2^ isoforms were detected (Fig. 4D). A band correlating with p60^MDM2^ was inconsistently observed in the blots for this cell line, and therefore, is not discussed further. There was a significant (*p* < 0.05) decrease in the expression of both the p75^MDM2^ and p90^MDM2^ isoforms after incubation of SK-N-MC cells with chelators and DNA-damaging agents relative to the control. However, the p21 levels were not significantly altered in response to chelators or DNA-damaging agents in this cell line (Fig. 3E). This lack of effect on p21 expression could be due to p75^MDM2^ (that may protect p21 from degradation) and p90^MDM2^ (that degrades p21; [56, 57]) being both significantly (*p* < 0.05) reduced to approximately the same extent (Fig. 4D). In addition, as found in MCF-7 cells (Fig. 4A), a 23 kDa band was also identified in SK-N-MC cells (Fig. 4D). As similarly observed for MCF-7 cells, a significant (*p* < 0.05) increase in the 23 kDa band was observed in these cells when incubated with chelators and CP, whereas a significant (*p* < 0.05) decrease was found after incubation with MC (Fig. 4D).

Overall, there was a close correlation between the expression of p21 and the p75^MDM2^ isoform in response to Fe chelators in three of the cell-types investigated (*i.e*., MCF-7 (*r* = +0.92), LNCaP (*r* = +0.92) and SK-MEL-28 (*r* = +0.81)). This observation suggested that one mechanism by which chelators may regulate p21 expression could potentially occur through regulating the expression of p75^MDM2^. However, MDM2 expression did not explain the response of the CFPAC-1 cells to chelators and DNA-damage, as this protein was not detected in this cell line. Hence, further experiments were conducted to examine the expression of another potential regulator of p21, namely ΔNp63.

### Expression of ΔNp63 is not altered by chelators or DNA-damaging agents in all cell-types investigated

The *p63* gene is a member of the *p53* gene family [82, 83]. It encodes multiple isoforms of p63 with a variety of functions, some of which have been shown to transactivate p53 target genes, including *p21* [84]. However, the ΔNp63 isoform lacks the transactivation domain and can act as a dominant negative regulator to inhibit transactivation by p53 and p63, leading to inhibition of target gene (*e.g*., *p21*) expression [60, 85, 86]. The over-expression of the ΔNp63 isoform has previously been reported as a negative regulator of cyclin-dependent kinase inhibitors [59], including p21 [58, 61]. Hence, ΔNp63 expression in response to the chelators was examined using an antibody specific for this particular isoform to determine if it was involved in the response of p21 to these agents (Fig. 3A–3E). Considering this, western blot analysis was used to assess the potential role of ΔNp63 in regulating p21 levels in all cell-types examined herein (*i.e*., MCF-7, LNCaP, SK-MEL-28, CFPAC-1 and SK-N-MC).

Two ΔNp63 bands were detected at approximately 63 kDa and 74 kDa in all cell lines assessed (Fig. Supplemental 1). These two bands have been previously reported as different isoforms of ΔNp63 [87, 88]. Densitometric analysis indicated that incubation with chelators or DNA-damaging agents had no significant (*p* > 0.05) effect on the expression of either the 63 or 74 kDa bands (Fig. Supplemental 1), this suggested that the alterations in p21 observed in these cell-types were independent of ΔNp63 expression.

### Nuclear localization of p21 following intracellular chelation and DNA-damage

**Figure 5:**
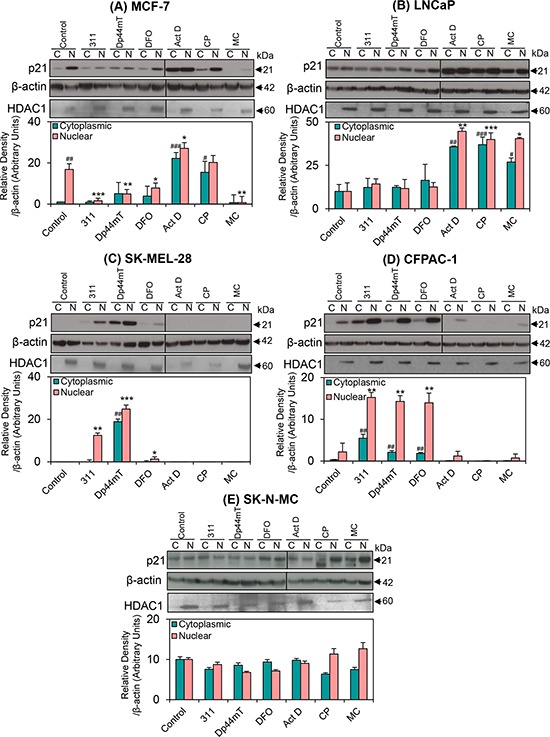
The effect of the chelators 311, Dp44mT, or DFO, and the DNA-damaging agents, Act D, CP or MC on the cytoplasmic and nuclear expression of p21 in five different tumor cell lines **A.** MCF-7; **B.** LNCaP; **C.** SK-MEL-28; **D.** CFPAC-1; and **E.** SK-N-MC cells. Cells were incubated for 24 h/37°C with the chelators, 311 (25 μM), Dp44mT (2.5 μM), DFO (250 μM), or the DNA-damaging agents, Act D (5 nM), CP (20 μM), or MC (30 μM). A vertical line appears on some blots to indicate the use of two gels. Indeed, in some experiments, due to hardware constraints, samples from one experiment were required to be run on 2 gels at the same time. These were then exposed equally under exactly the same conditions at the same time. The blots are typical of 3–6 independent experiments, while the densitometric analysis is mean ± SD (3–6 experiments). Relative to untreated control (cytoplasmic fraction): #*p* < 0.05, ##*p* < 0.01, ###*p* < 0.001. Relative to untreated control (nuclear fraction): *p < 0.05, **p < 0.01, ***p < 0.001.

Next, considering that the nuclear localization of p21 is crucial for its inhibitory function [45], cytoplasmic and nuclear fractionation studies were conducted to assess the intracellular location of the p21 protein following incubation of cells with chelators or DNA-damaging agents (Fig. 5A–5E). Histone deacetylase 1 (HDAC1) was also assessed to check the purity of the nuclear fractions and was shown to be appropriate in all experiments (Fig. 5A–5E).

Examination of MCF-7 cells incubated with control medium alone demonstrated that there was markedly and significantly (*p* < 0.01) greater p21 levels in the nuclear (N) fraction as compared to the cytoplasmic (C) fraction (Fig. 5A). Moreover, relative to the control, nuclear p21 levels were significantly (*p* < 0.001–0.05) reduced by all chelators, while cytoplasmic p21 was not significantly (*p* > 0.05) altered by these agents (Fig. 5A). This observation reflects the reduction in total p21 protein levels observed previously relative to the control (Fig. 3A). The decrease of nuclear p21 in response to these chelators is consistent with previous studies in this cell line [71]. Assessing MCF-7 cells treated with Act D, there was a significant (*p* < 0.001–0.05) increase in p21 protein present in both cytoplasmic and nuclear fractions relative to the control (Fig. 5A). Cells incubated with CP demonstrated a significant (*p* < 0.05) increase in cytoplasmic, but not nuclear p21, when compared to the controls. In contrast to Act D and CP, incubation with MC led to a significant (*p* < 0.01) decrease in nuclear levels of p21 relative to the control (Fig. 5A). This finding was consistent with the effects of MC on total p21 protein expression (Fig. 3A).

In contrast to MCF-7 cells (Fig. 5A), untreated LNCaP cells expressed p21 at approximately equal levels in both the cytoplasm and nucleus (Fig. 5B). Moreover, following incubation of these cells with 311, Dp44mT or DFO, there was no significant (*p* > 0.05) change in nuclear or cytoplasmic p21 levels, which is in agreement with the total p21 levels (Fig. 3B). On the other hand, a significant (*p* < 0.001–0.05) up-regulation of p21 in both the cytoplasm and nucleus was observed in LNCaP cells incubated with all DNA-damaging agents (Fig. 5B) that was also in correlation with total p21 expression (Fig. 3B).

Of all the cell lines utilized, SK-MEL-28 was the only cell line, which had no detectable p21 expression in the control cytosolic or nuclear fractions (Fig. 5C). However, following incubation of these cells with 311 or DFO, nuclear p21 was significantly (*p* < 0.01–0.05) increased, while cytoplasmic p21 levels were not markedly affected. On the other hand, SK-MEL-28 cells incubated with Dp44mT demonstrated significantly (*p* < 0.001–0.01) higher levels of p21 protein in both the cytoplasmic and nuclear fractions relative to the control (Fig. 5C). The DNA-damaging agents did not significantly (*p* > 0.05) affect p21 expression in the cytoplasmic or nuclear fractions of this cell line. These data were in agreement with total p21 levels (Fig. 3C).

In CFPAC-1 cells incubated with control medium alone, p21 expression was greater, but not significantly (*p* > 0.05) different in the nuclear fraction relative to the cytoplasmic fraction (Fig. 5D). As observed with total p21 levels (Fig. 3D), when CFPAC-1 cells were incubated with chelators, there was a significant (*p* < 0.01) increase in p21 protein levels in both the cytoplasmic and nuclear fractions compared with the controls. In contrast, after incubation with DNA-damaging agents, there was no significant (*p* > 0.05) change in cytoplasmic or nuclear p21 levels.

Data obtained from SK-N-MC cells showed relatively equal amounts of p21 in both the cytoplasmic and nuclear fractions, regardless of chelator treatment (Fig. 5E). Furthermore, no significant (*p* > 0.05) alterations were observed with the DNA-damaging agents compared to the untreated control (Fig. 5E) in correlation with the total p21 levels (Fig. 3E).

**Figure 6:**
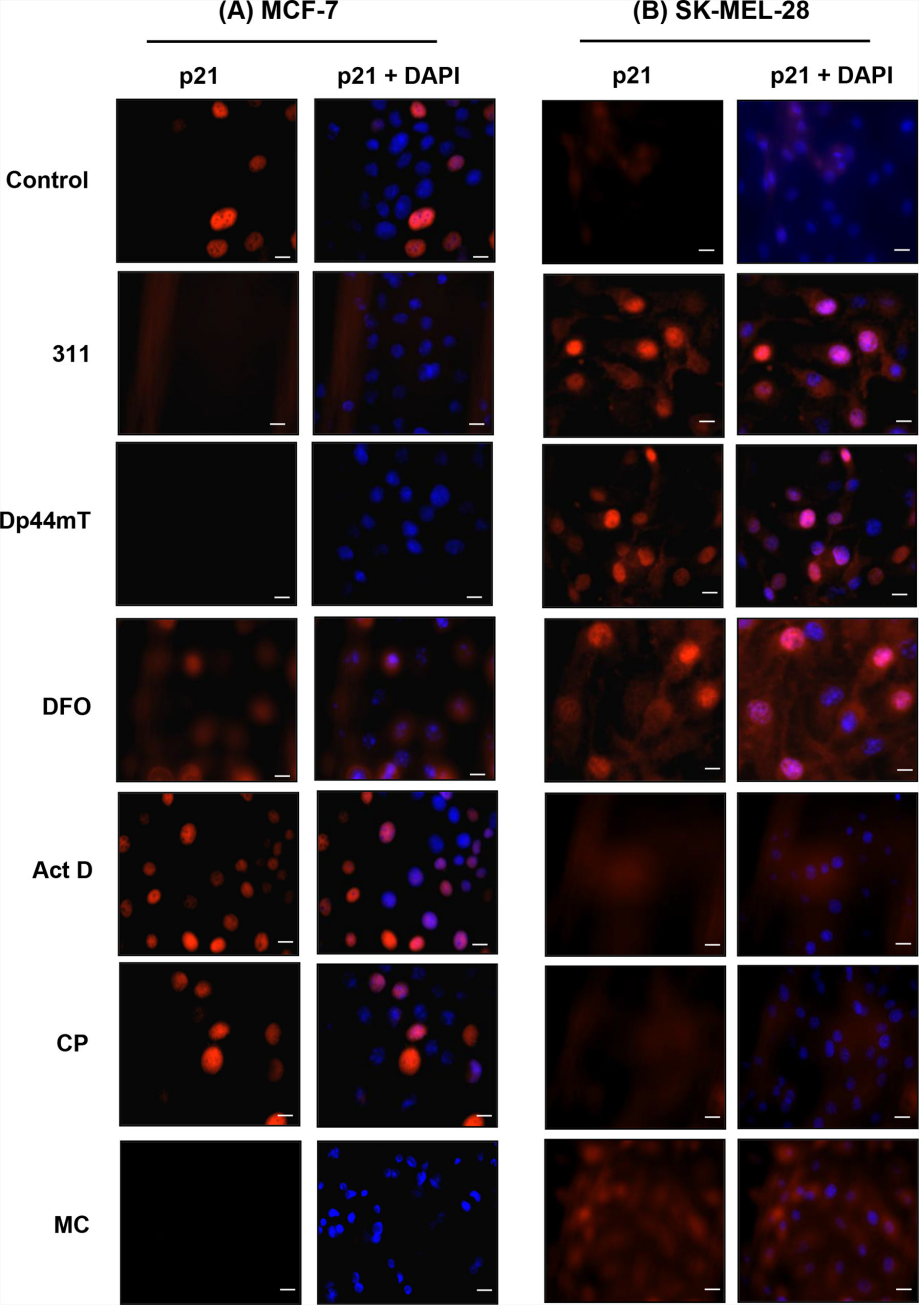
Immunofluorescence studies examining the cellular distribution of p21 observed in: **A.** MCF-7 cells, or **B.** SK-MEL-28 cells, in response to chelators or DNA-damaging agents Cells were incubated for 24 h/37°C with the chelators, 311 (25 μM), Dp44mT (2.5 μM), DFO (250 μM), or the DNA-damaging agents, Act D (5 nM), CP (20 μM), or MC (30 μM). Photographs were taken from cells incubated with primary p21 antibody and Alexa Fluor^®^ 594 fluorescent dye conjugated secondary antibody and 4*'*, 6-diamidino-2-phenylindole (DAPI). An electronic merge of both photos was used to demonstrate the nuclear localization of p21. The photographs are typical of three experiments. The scale bar in each image represents 20 μm.

Immunofluorescence studies were then conducted to further assess the cellular distribution of p21 in the MCF-7 and SK-MEL-28 cell lines (Fig. 6A–6B). These two cell lines were chosen as the focus for these experiments because of their distinct expression of p21 protein in response to Fe chelator treatment, namely a reduction in MCF-7 cells (Figs. 3A, 5A) and an increase in SK-MEL-28 cells (Figs. 3C, 5C). Fig. 6A confirms that the overall levels of p21 in the MCF-7 cell line decrease upon treatment with chelators. These results also demonstrate the increased levels and accumulation of p21 in the nucleus of MCF-7 cells incubated with the DNA-damaging agents, Act D and CP, which was in agreement with the fractionation results in Fig. 5A. On the other hand, there was a marked decrease in the intensity of p21 expression after incubation with MC (Fig. 6A) and this was consistent with the western analysis (Fig. 3A, 5A). In agreement with the fractionation results (Fig. 5C), immunofluorescence studies demonstrated nuclear accumulation of p21 in SK-MEL-28 cells in response to the chelators, but not in cells treated with the DNA-damaging agents. In summary, there was a marked difference in the expression levels and cellular distribution of p21 depending upon the cell-type examined and also the response of these cells to the chelators.

## DISCUSSION

It has been reported that some cancer cells have elevated p21 levels, while maintaining the ability to rapidly proliferate [89–91]. In fact, cytoplasmic p21 expression is common in human malignancies and correlates positively with aggressive tumors and poor prognosis [92–94]. Considering that Fe is a new metabolic target for inhibiting cancer cell proliferation, with several Fe chelators already entering clinical trials [9, 15, 16], we assessed the effect of three of these agents compared to DNA damaging agents on p21 expression. This is important because Fe is known to be involved in the progression of cells through the cell cycle by influencing the expression of a variety of molecules involved in cell cycle control, such as p21 and p53 [8–11]. These studies were conducted in five different cancer cell lines, two of which were expressing wild-type p53 (MCF-7 and LNCaP); two expressing mutant forms of p53 (SK-MEL-28 and CFPAC-1); and one cell line that was p53 null (SK-N-MC; Table 1) [65–69].

Initial experiments demonstrated that chelators were able to markedly up-regulate *p21* mRNA levels, in all of the cell-types examined (Fig. 2A–2E), regardless of their p53 status. However, the expression of p21 protein in response to chelator treatment did not correspond to its mRNA levels in all cell lines examined. In fact, incubation of cells with chelators resulted in decreased total p21 protein in MCF-7 cells (Fig. 3A), whereas there was an increase in p21 expression in SK-MEL-28 (Fig. 3C) and CFPAC-1 cells (Fig. 3D). On the other hand, there was no significant change in p21 protein levels after incubation of LNCaP or SK-N-MC cells with the chelators (Fig. 3B, 3E).

Despite that both MCF-7 and LNCaP cells have wild-type p53 expression (Table 1), their responses to chelators were markedly different, with MCF-7 cells having reduced p21 expression, while there was no significant change in total p21 protein in LNCaP cells. This observation indicates that the effect of chelators on p21 expression may not involve p53 and could be occurring through other mechanisms. This was further demonstrated by examining CFPAC-1 and SK-MEL-28 cells, where the chelators were able to markedly increase p21 levels (Fig. 3C, 3D), despite both these cells having mutated p53 (Table 1). Collectively, these data suggest that the chelator-induced regulation of p21 does not correlate with p53 status and is cell line-dependent.

In contrast to p21 protein levels, *p21* mRNA expression was consistently increased in each of the cell lines examined in response to chelation (Fig. 2A–2E). This indicates that p21 translation may potentially be inhibited in some of these cells and warrants further investigation. Alternatively, p21 could be regulated at the post-transcriptional level by protein degradation [95]. In fact, Fu and Richardson [70] examined the down-regulation of p21 following Fe-depletion in MCF-7 cells and demonstrated that it was due to: (**1**) inhibited translocation of *p21* mRNA from the nucleus to cytosolic translational machinery; and (**2**) induction of ubiquitin-independent proteasomal degradation. Whether any of these mechanisms are responsible for the failure of the increased *p21* mRNA to be translated to protein in LNCaP and SK-N-MC cells after incubation with chelators is not known.

Upon treatment of the five cell-types assessed herein with DNA-damaging agents, *p21* mRNA expression was significantly up-regulated in all cell lines examined, except after incubation of SK-MEL-28 and CFPAC-1 cells with Act D (Fig. 2A–2E). The increase in *p21* mRNA levels occurred irrespective of p53 status and suggested that the DNA-damaging agents could up-regulate *p21* mRNA by a p53-independent mechanism. A paradoxical increase of *p21* mRNA (Fig. 2A) and decrease in protein expression (Fig. 3A) was observed in response to the DNA-damaging agent, MC, in MCF-7 cells. This trend was also observed after incubation of SK-MEL-28 and CFPAC-1 cells with CP or MC (Fig. 2C, 2D and 3C, 3D). In the case of MC, it has been reported that this agent also inhibits the transcription of ribosomal RNA, which could significantly decrease protein translation in MCF-7 cells [96] and may explain the decreased p21 protein levels observed with this agent.

Recently, NDRG1 was found to up-regulate *p21* mRNA and protein in PC3 and DU145 prostate cancer cells and H1299 lung cancer cells, independently of p53 [41]. NDRG1 functions as a metastasis suppressor in various cancers (for review see [97]) and has been shown to be up-regulated in response to Fe chelator treatment in a variety of cell-types [12, 41]. The increased expression of NDRG1 in response to Fe-deprivation in all the cell lines used in this investigation agreed with that previously observed in prostate and lung cancer cell lines [41]. However, NDRG1 did not directly correlate with p21 protein expression in MCF-7, LNCaP, or SK-N-MC cells, suggesting that alternative, NDRG1-independent pathways could be involved in the chelator-mediated effects on p21 in these cell-types.

As previously mentioned, MDM2 has been reported to regulate the expression of p21 independently of p53, by directly promoting its proteasomal degradation [56, 57]. The many MDM2 isoforms arise through proteolytic cleavage, post-translational modification, or alternative splicing [78, 79]. Considering this, Cheng and Cohen [98] reported that the p90^MDM2^ isoform is an un-conjugated full-length MDM2 protein, whereas p75^MDM2^ is an *N*-terminal truncated protein isoform. The over-expression of p75^MDM2^, which cannot bind p53, has been found to interfere with the ability of p90^MDM2^ to promote p53 degradation [62]. Several isoforms of MDM2 were identified in the cells used in this investigation (Fig. 4A–4D). Of particular interest, the p75^MDM2^ isoform showed a close correlation to the expression of p21 in three of the five cell lines examined under all treatment conditions. In this case, the chelators appear to have activated the *MDM2* promoter independently of p53, resulting in the generation of the p75^MDM2^ isoform, which may in turn interfere with the ability of p90^MDM2^ to promote p21 degradation [56]. Besides its effects on p53 and p21, full-length MDM2 has a number of other oncogenic functions in cancer cells, which include the alteration of cell proliferation, apoptosis, invasion and metastasis [99, 100]. However, the exact mechanisms by which chelators modulate MDM2 function are unknown and further studies are required to elucidate how, and why, particular isoforms are affected in response to Fe chelation.

Another member of the p53 family, p63, has also been found to contain multiple isoforms with various activities [83, 84]. The TAp63 isoform, which contains the transactivation domain, has transcriptional activity and transactivates p53 target genes such as *p21* and *MDM2* [83, 101]. The ΔNp63 isoform lacks the transactivation domain and its over expression has previously been reported as a negative regulator of p21 [58, 60, 61]. However, this study indicated that the alterations in p21 observed in these cell-types were independent of ΔNp63 expression, with no changes in ΔNp63 detected in response to the chelators.

Finally, this investigation demonstrated that for SK-MEL-28 and CFPAC-1 cells there was increased nuclear localization of p21 compared to cytosolic levels in response to chelators. Given the inhibitory effects of p21 when localized to the nucleus [45], which include its cdk inhibitory function and promotion of cellular senescence, the nuclear accumulation of p21 observed could explain how chelators lead to G_1_/S arrest in these cell-types [21, 25]. Considering that p21 can aid in cell cycle progression by stabilising the interactions between cyclins and cdks under certain conditions [45, 49, 50], the decrease in the endogenously high levels of p21 in MCF-7 cells in response to chelators may partially explain their anti-proliferative activity in this cell line [23].

In conclusion, this study examined the mechanisms involved in the chelator-mediated regulation of p21 in five tumor cell lines. Overall, the results indicated that chelators regulate p21 expression independently of p53 status and in a cell line-dependent manner. Furthermore, we identified that the 75 kDa isoform of MDM2 (p75^MDM2^) closely resembled the expression of p21 in response to Fe chelators in three of the five cell lines examined, suggesting that MDM2 may be involved in the regulation of p21 protein in response to these agents.

## METHODS

### Cell culture

Cells were purchased from the American Type Culture Collection (ATCC; MD, USA). These include: the human breast cancer cell line, MCF-7; the androgen-sensitive human prostate adenocarcinoma cells, LNCaP; the human melanoma cell line, SK-MEL-28; the human pancreatic ductal adenocarcinoma cell line, CFPAC-1; and the neuroepithelioma cell line derived from a supra orbital brain tumor, SK-N-MC. The MCF-7, SK-MEL-28 and SK-N-MC cell lines were maintained in Minimal Essential Medium (MEM; Invitrogen; CA, USA). The CFPAC-1 cell line was maintained in Dulbecco's Modified Eagle Medium (DMEM; Invitrogen), whereas the LNCaP cell line was maintained in Roswell Park Memorial Institute medium (RPMI; Invitrogen). All media was supplemented with 10% fetal calf serum (FCS; Sigma Aldrich; MO, USA), 100 μg/mL penicillin/streptomycin/glutamine (Invitrogen), 0.1 mM non-essential amino acids (Invitrogen), 1 mM sodium pyruvate (Invitrogen) and 0.28 ng/mL Fungizone™ (Invitrogen). Cells were grown at 37°C in 5% CO_2_ in a cell culture incubator.

### Cell treatments

Cells were treated with various chelators, including: DFO (Novartis; Basel, Switzerland) at 250 μM, 311 at 25 μM, Dp44mT at 2.5 μM, and the DNA-damaging agents, Act D at 5 nM (Sigma Aldrich; MO, USA); CP at 20 μM (Sigma Aldrich); MC at 30 μM (Sigma Aldrich) for a period of 24 h/37°C. The ligands, 311 and Dp44mT were synthesized and characterized by standard methods [35, 102]. All agents were dissolved directly in culture media excluding 311 and Dp44mT, which were initially dissolved in dimethyl sulfoxide (DMSO; Sigma Aldrich) and then diluted in culture media (final DMSO concentration was ≤ 0.25% (v/v)).

### RNA isolation and RT-PCR

RNA was isolated using TRI Reagent Solution, following the manufacturer's protocol (Applied Biosystems; CA, USA). Reverse transcription-polymerase chain reaction (RT-PCR) was performed according to the methodology described in [70] using the primers provided in Supplementary Table 1.

### Western blot analysis

Protein was extracted from either whole cell lysates, as described previously [9], or nuclear and cytoplasmic fractions using the NE-PER Nuclear and Cytoplasmic Extraction Reagent Kit (Pierce; IL, USA). Western blot analysis was performed *via* established methods [9]. Primary antibodies used were against p21 (1:1000; Cat.#: 2947S; Cell Signaling Technology, MA, USA); NDRG1 (1:2000; Cat.#: ab37897; Abcam, Cambridge, UK); HDAC1 (1:1000; Cat.#: 2062S; Cell Signaling); MDM2-SMP14 (1:1000; Cat.#: sc-965; Sigma Aldrich); and ΔNp63 (1:400; Cat.#: sc-8609; Santa Cruz Biotechnology; CA, USA). The secondary antibodies used were all conjugated to horseradish peroxidase (HRP) and include: anti-rabbit IgG, anti-goat IgG and anti-mouse IgG (1:10,000; Cat.#: A0545, A5420, A9917; Sigma Aldrich). β-actin (1:10,000; Cat.#: A5316; Sigma Aldrich) was used as a protein-loading control.

In some experiments, due to electrophoresis hardware constraints, samples from one experiment were required to be run on 2 gels at the same time. These were then exposed equally under exactly the same conditions at the same time. When this was done, a vertical line is shown in the gel (*i.e*., see Fig. 5).

### Immunofluorescence

Immunofluorescence was performed by established methods, as described previously [10]. Briefly, cells were seeded onto coverslips and fixed in ice-cold methanol for 15 min and then permeabilized with PBS containing 0.25% Triton X-100 for 10 min at room temperature. The cells were then incubated with the p21 primary antibody (1:400) above, overnight at 4°C. This procedure was then followed by incubation with the secondary antibody, namely anti-rabbit Ab conjugated to Alexa Fluor^®^ 594 fluorescent dye (1:1000; Cat.#: A11012; Cell Signaling) for 1 h at room temperature. The coverslips were mounted onto slides using an anti-fade mounting solution containing 4′,6-diamidino-2-phenylindole (DAPI; Invitrogen). The slides were then analysed using a Zeiss Axio Observer.Z1 fluorescent microscope and images were taken and analysed using Axiovision software (Carl Zeiss AG, Oberkochen, Germany).

### Densitometry and statistical analysis

Densitometry was performed using Quantity One software (Bio-Rad, Hercules, CA) and normalised using the relative β-actin loading control. Results are typical of three to six independent experiments and presented as mean ± S.D. Experimental data were compared using the Student's *t*-test. Results were considered statistically significant when *p* < 0.05.

## SUPPLEMENTARY FIGURE AND TABLE


