# Supplementary material for: Differential targeting of the cyclin-dependent kinase inhibitor, p21CIP1/WAF1, by chelators with anti-proliferative activity in a range of tumor cell-types
Source: Oncotarget. 2015 Aug 22;6(30):29694–711. doi: 10.18632/oncotarget.5088 (PMC4745756; doi:10.18632/oncotarget.5088)
Supplement: Supplementary file 1 [file oncotarget-06-29694-s001.pdf]

SUPPLEMENTARY FIGURE AND TABLE

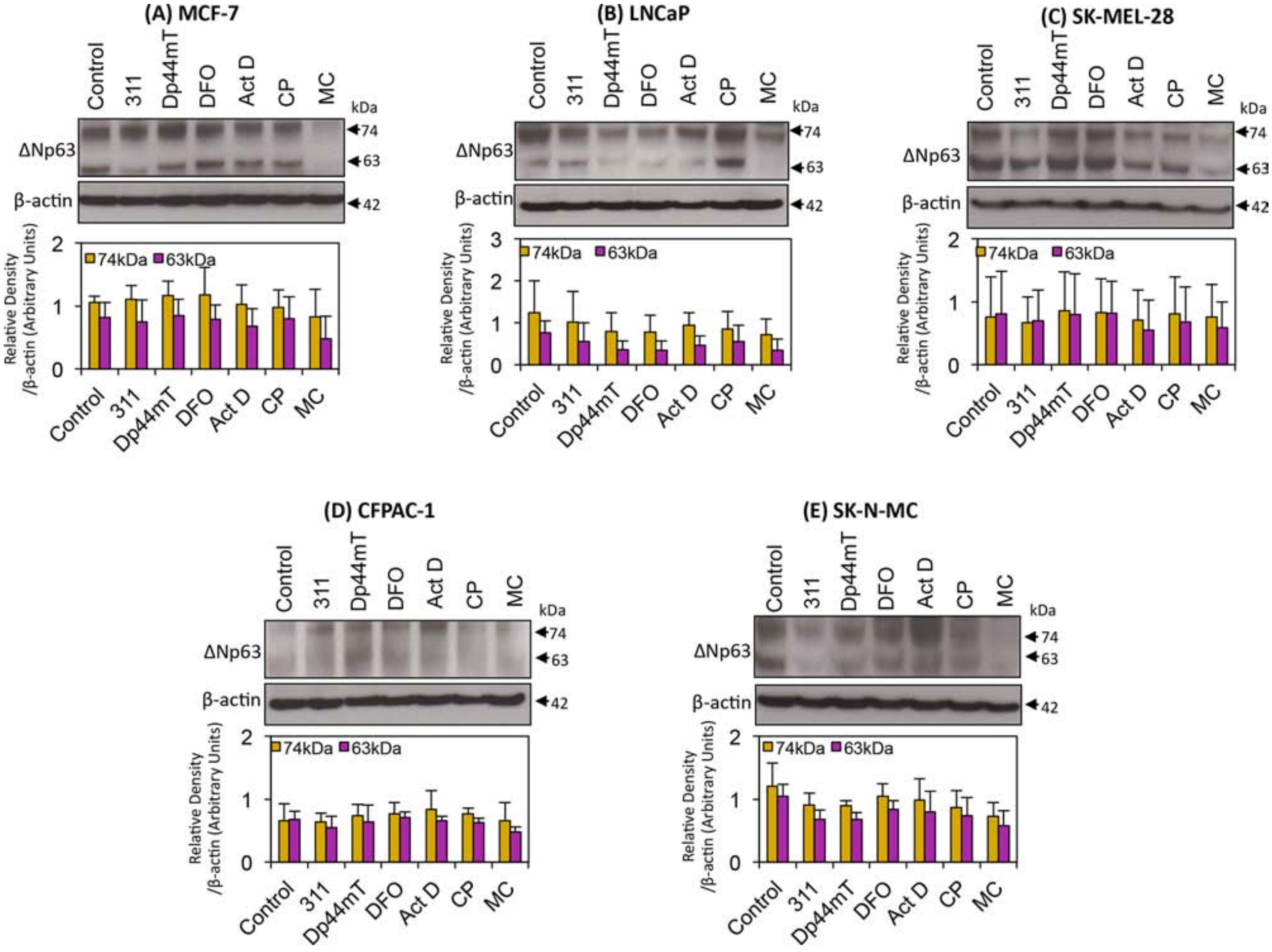

Supplementary Figure S1: The effect of the chelators 311, Dp44mT, or DFO, and the DNA-damaging agents, Act D, CP or MC on the expression of ΔNp63 protein in five different tumor cell lines. A. MCF-7; **B.** LNCaP; **C.** SK-MEL-28; **D.** CFPAC-1; and **E.** SK-N-MC cells. Cells were incubated for 24 h/37°C with the chelators, 311 (25 µM), Dp44mT (2.5 µM), DFO (250 µM), or the DNA-damaging agents, Act D (5 µM), CP (20 µM), or MC (30 µM). The blots are typical of 3 independent experiments, while the densitometric analysis is mean ± SD (3 experiments).

Supplementary Table S1: Primer sequences used for RT-PCR.

Gene	Final Conc (nM)	Sequence (5′-3′)		Product size (bp)
		Fwd	Rev	
CDKN1A (p21)	200	GACTGTGATGCGCTAATGG	GGTAGAAATCTGTCATGCTGGT	358
NDRG1	200	GGATCAGTTGGCTGAAAT	ATCTTGAGTAGGGTGGTCTT	513
β-actin	200	CCCGCCGCCAGCTCACCATGG	AAGGTCTCAAACATGATCTGGGTC	397
